# Structural insights into the ligand binding and G_i_ coupling of serotonin receptor 5-HT_5A_

**DOI:** 10.1038/s41421-022-00412-3

**Published:** 2022-05-24

**Authors:** Yangxia Tan, Peiyu Xu, Sijie Huang, Gong Yang, Fulai Zhou, Xinheng He, Honglei Ma, H. Eric Xu, Yi Jiang

**Affiliations:** 1 The CAS Key Laboratory of Receptor Research, Shanghai Institute of Materia Medica, Chinese Academy of Sciences, Shanghai, China; 2 School of Life Science and Technology, ShanghaiTech University, Shanghai, China; 3 University of Chinese Academy of Sciences, Beijing, China; 4 State Key Laboratory of Cellular Stress Biology, School of Life Sciences, Xiamen University, Xiamen, Fujian China; 5 State Key Laboratory of Microbial Technology, Shandong University, Qingdao, Shandong China; 6 Lingang Laboratory, Shanghai, China

**Keywords:** Cryoelectron microscopy, Hormone receptors

## Abstract

5-hydroxytryptamine receptor 5A (5-HT_5A_) belongs to the 5-HT receptor family and signals through the G_i/o_ protein. It is involved in nervous system regulation and an attractive target for the treatment of psychosis, depression, schizophrenia, and neuropathic pain. 5-HT_5A_ is the only G_i/o_-coupled 5-HT receptor subtype lacking a high-resolution structure, which hampers the mechanistic understanding of ligand binding and G_i/o_ coupling for 5-HT_5A_. Here we report a cryo-electron microscopy structure of the 5-HT_5A_–G_i_ complex bound to 5-Carboxamidotryptamine (5-CT). Combined with functional analysis, this structure reveals the 5-CT recognition mechanism and identifies the receptor residue at 6.55 as a determinant of the 5-CT selectivity for G_i/o_-coupled 5-HT receptors. In addition, 5-HT_5A_ shows an overall conserved G_i_ protein coupling mode compared with other G_i/o_-coupled 5-HT receptors. These findings provide comprehensive insights into the ligand binding and G protein coupling of G_i/o_-coupled 5-HT receptors and offer a template for the design of 5-HT_5A_-selective drugs.

## Introduction

**Fig. 1:**
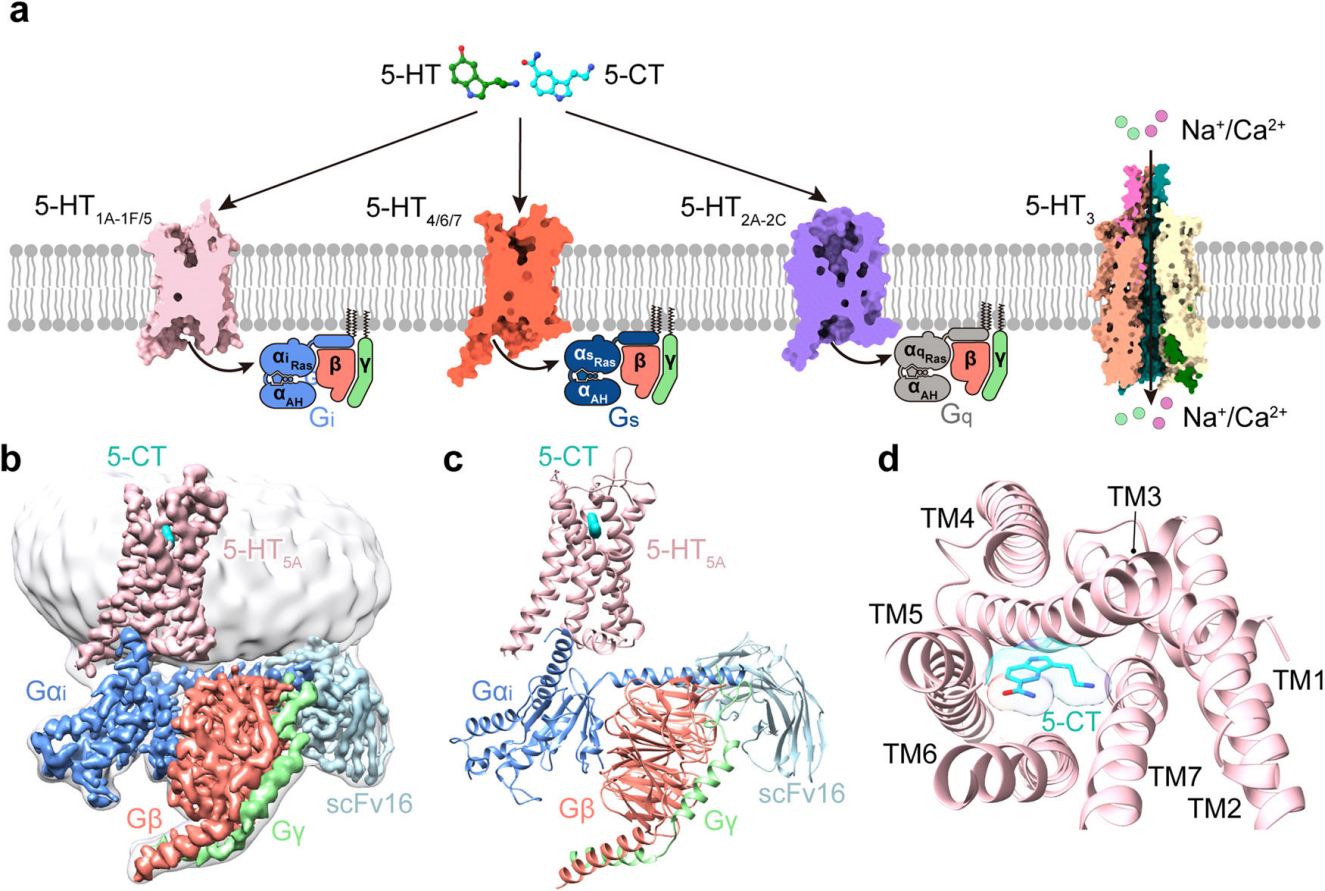
Cryo-EM structure of the 5-CT–5-HT_5A_–G_i_–scFv16 complex. **a** Schematic illustration of 5-HT receptors coupling to distinct G proteins upon stimulation by 5-HT and 5-CT. 5-HT_1_ and 5-HT_5_ belong to G_i/o_-coupled 5-HT receptors. 5-HT_1_ subfamily includes 5-HT_1A_, 5-HT_1B_, 5-HT_1D_, 5-HT_1E_, and 5-HT_1F_. Different from other 5-HT receptor subtypes, 5-HT_3_ is an ion channel. **b**, **c** Orthogonal views of the density map (**b**) and model (**c**) for the 5-CT**–**5-HT_5A_**–**G_i_**–**scFv16 complex. The EM density of 5-CT is shown as cyan surface presentation. **d** The extracellular view of the 5-HT_5A_. The complex is colored by subunits. Light pink, 5-HT_5A_; cyan, 5-CT; blue, Gα_i_; salmon, Gβ; light green, Gγ; light blue, scFv16.

5-hydroxytryptamine (5-HT) receptors are widely expressed in the central and peripheral nervous systems and are involved in a variety of psychiatric disorders. They are one of the most promising drug targets for the treatment of nervous system diseases^1^. There are seven distinct types (5-HT_1–7_), comprised of 14 subtypes in the 5-HT receptor family, of which 13 are G protein-coupled receptors (GPCRs) (Fig. 1a). So far, 26 structures of 5-HT receptors have been reported, including crystal structures of 5-HT_1B_^2,3^, 5-HT_2A_^4,5^, 5-HT_2B_^6–9^, and 5-HT_2C_^10^, as well as cryo-electron microscopy (cryo-EM) structures of all members of 5-HT_1_, including 5-HT_1A_^11^, 5-HT_1B_^12^, 5-HT_1D_^11^, 5-HT_1E_^11^, and 5-HT_1F_^13^, in complex with G_i/o_ protein. These structures provide a basis for understanding ligand recognition and functional regulation of these 5-HT receptors. Besides 5-HT_1_, 5-HT_5_ is another type of G_i/o_-coupled 5-HT receptor and also remains the last type of G_i/o_-coupled 5-HT receptor without a reported structure.

The 5-HT_5_ subfamily consists of two members, designated as 5-HT_5A_ and 5-HT_5B_, which share 69% sequence identity with each other and have 23%–34% homology with other 5-HT receptors^14^. Of note, 5-HT_5B_ is the first example of a brain-specific receptor that is absent in humans, of which the coding sequence is interrupted by stop codons^14,15^. Thus, 5-HT_5A_ stands out as the only 5-HT_5_ subtype expressed in human brain regions, including the cerebral cortex, hippocampus, and raphe nuclei^16,17^. 5-HT_5A_ shows an antinociceptive role and is involved in the regulation of memory, learning, and food intake^18,19^. Its specific ligands have shown potential in the treatment of psychosis, depression, schizophrenia, and neuropathic pain^20^. Thus, the development of 5-HT_5A_-selective drugs will offer a new opportunity for the treatment of these nervous system diseases.

However, the selective ligands for 5-HT_5A_ are still lacking. 5-Carboxamidotryptamine (5-CT) is a synthetic agonist for 5-HT_5A_ and also activates other G_i/o_-coupled 5-HT receptors with distinct affinities. It shows a moderate affinity for 5-HT_5A_ with a *pK*_i_ value of 7.7 and displays high affinities for 5-HT_1A_, 5-HT_1B_, and 5-HT_1D_ (*pK*_i_ = 8.9–9.0)^21–25^. In contrast, 5-CT displays negligible affinities for 5-HT_1E_ (*pK*_i_ = 5.4) and 5-HT_1F_ (*pK*_i_ = 6.1) (https://pdsp.unc.edu/pdspweb/)^26,27^. SB699551 and ASP5736 stand out as two selective antagonists, which have been widely used for functional studies of 5-HT_5A_^20,28^. The availability of the structure of ligand-bound 5-HT_5A_ may accelerate the design of 5-HT_5A_-targeting drugs by providing an accurate structure template.

In this study, we report the structure of G_i_-coupled 5-HT_5A_ complex bound to 5-CT at a resolution of 3.1 Å. This structure clarified the feature of 5-CT recognition by 5-HT_5A_ and identified a determinant for 5-CT affinities against G_i/o_-coupled 5-HT receptors, thus providing a rationale for designing drugs targeting 5-HT_5A_. Structural comparison of the 5-HT_5A_–G_i_ with other G_i/o_-coupled 5-HT receptor complexes deepens our understanding of the mechanism underlying ligand recognition and G_i/o_ coupling.

## Results

### Cryo-EM structure of the 5-CT–5-HT_5A_–G_i_–scFv16 complex

We used the full-length human 5-HT_5A_ for structural studies. A BRIL was fused to the N-terminus of 5-HT_5A_ to improve expression. The NanoBiT tethering strategy was applied to stabilize the 5-HT_5A_–G_i_ complex, which had been widely used in the structure determination of several GPCR–G protein complexes^29–31^ (Supplementary Fig. S1). The C-terminus of the receptor and the Gβ_1_ subunit were connected to the LgBiT and HiBiT, respectively. A dominant-negative form of the human Gα_i1_ mutant containing four mutations (S47N, G203A, E345A, and A326S), referred to as Gα_i1(4DN)_, was applied^32^. The 5-HT_5A_–G_i_ complex was assembled by co-expressing the engineered receptor with Gα_i1(4DN)_, Gβ_1_, Gγ_2_ subunits, and scFv16 in High Five (Hi5) cells in the presence of 5-CT.

The structure of the 5-CT–5-HT_5A_–G_i_–scFv16 complex was determined with an overall resolution of 3.1 Å (Fig. 1b, c; Supplementary Fig. S2 and Table S1). The high-quality density maps are clear for modeling 5-HT_5A_ from residue 31 to residue 353, with the exception of residues 237–275 in the intracellular loop 3 (ICL3). The majority of the residue side chains in the seven-transmembrane helical domain (TMD), three extracellular loops (ECL1–ECL3), and two ICLs (ICL1 and ICL2) of 5-HT_5A_ were well-defined. 5-CT, scFv16, and the three subunits of G_i_ protein are also well-fitted in the EM map. The entire model provides detailed structural information on the 5-CT-binding pocket and 5-HT_5A_–G_i_ interaction interface (Fig. 1c, d; Supplementary Fig. S3).

### The recognition of G_i/o_-coupled 5-HT receptors by agonists

**Fig. 2:**
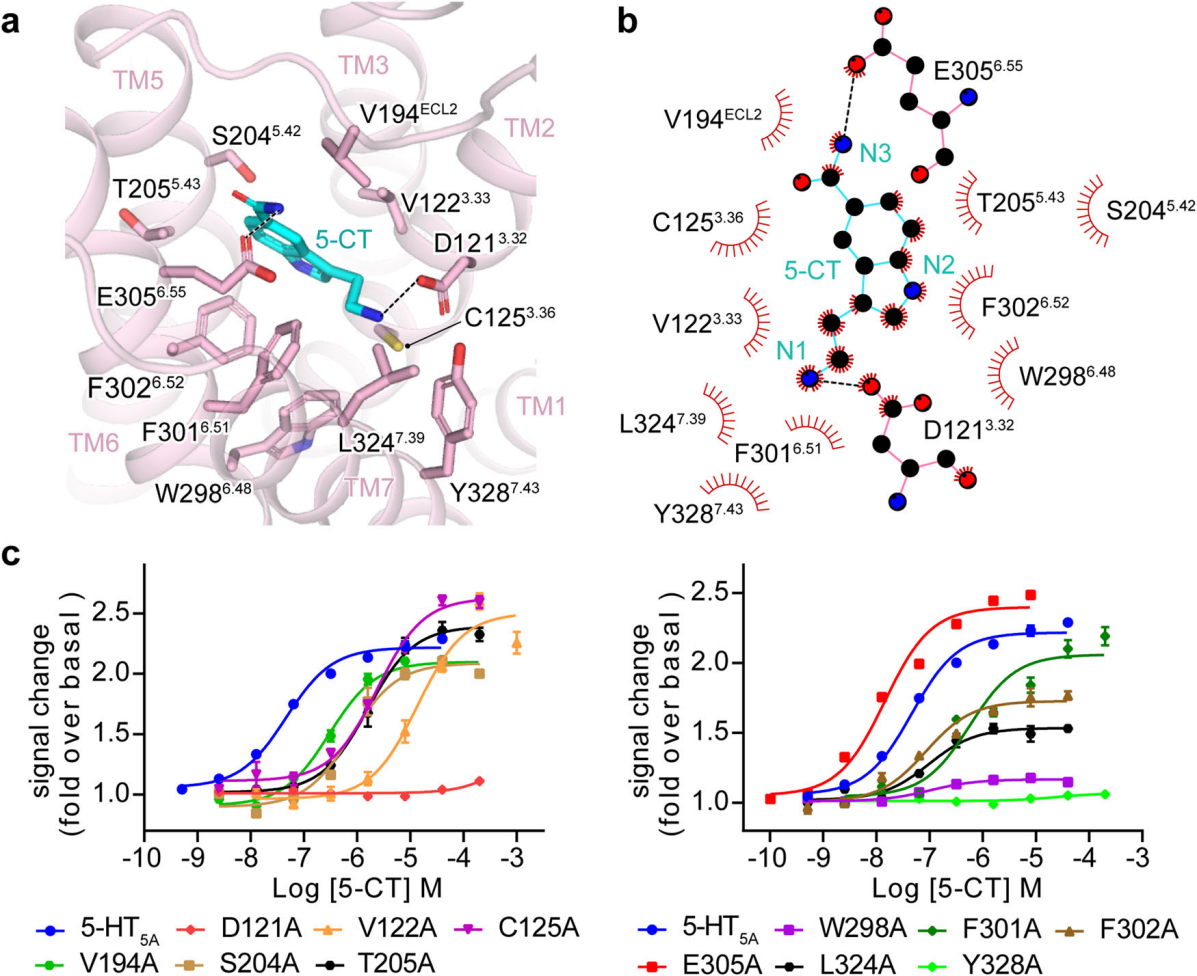
The 5-CT-binding pocket of 5-HT_5A_. **a** Detailed interactions of 5-CT with residues in the binding pocket of 5-HT_5A_. **b** 2D schematic representation of interactions between 5-CT and residues in the ligand-binding pocket of 5-HT_5A_, analyzed by LigPlot^+^ program. The polar interactions are indicated as black dashed lines. **c** Effects of alanine mutation of pocket residues of 5-HT_5A_ on 5-CT-induced G_i_ protein recruitment. Three independent NanoBiT assays in triplicates were performed. Each data point presents mean ± SEM from a representative experiment.

The binding pocket in 5-HT_5A_ is largely overlapped with that in other G_i/o_-coupled 5-HT receptors, sharing 11 of 16 identical residues. 5-CT is embedded deep into the pocket constituted by TM3, ECL2, and TM5–TM7 of 5-HT_5A_ (Fig. 2a). Compared with other ligands bound to G_i/o_-coupled 5-HT receptors, 5-CT adopts a similar binding pose in 5-HT_5A_ (Supplementary Fig. S4a). Its indole scaffold is anchored through a salt bridge between its positively charged nitrogen at the 3-aminoethyl group and the carboxylate of D121^3.32^ (Fig. 2a, b). This salt bridge is highly conserved across ligand-bound 5-HT receptors with known structures (Supplementary Fig. S4b). Mutating D121^3.32^ to alanine abolished the 5-CT-induced 5-HT_5A_ activation, highlighting its importance to 5-CT activity (Fig. 2c). In addition, the side chain of D121^3.32^ is further stabilized by an intramolecular hydrogen bond between D121^3.32^ and Y328^7.43^, which is supported by the alanine mutagenesis data. On the other side, the nitrogen at the 5-carboxamide of 5-CT forms a hydrogen bond with the side chain of E305^6.55^. Besides polar interactions, the indole scaffold of 5-CT tightly packs against a hydrophobic cleft comprising side chains of V122^3.33^, F301^6.51^, F302^6.52^, and L324^7.39^. These hydrophobic residues substantially contribute to 5-CT-induced 5-HT_5A_ activation (Fig. 2c; Supplementary Table S2).

It has been thought that the ligand-binding pocket of each G_i/o_-coupled 5-HT receptor is comprised of two subpockets, the orthosteric binding pocket (OBP) and the extended binding pocket (EBP)^2,8^. OBP of G_i/o_-coupled 5-HT receptors locates deep into the core of the TMD pocket, whereas the EBP approaches the extracellular surface of the entire binding pocket (Supplementary Fig. S4a). The 11 conserved residues in the binding pocket across G_i/o_-coupled 5-HT receptors are located in the OBP, including D^3.32^, C^3.36^, T^3.37^, I^4.56^, S^5.42^, T^5.43^, A^5.46^, W^6.48^, F^6.51^, F^6.52^, and Y^7.43^ (Supplementary Fig. S4b), of which D^3.32^ is thought critical to ligand binding for 5-HT and other monoamine receptors by forming a conserved salt bridge with the basic cyclic amine of ligands^2^. The featured benzene ring of the ligand is surrounded by conserved hydrophobic residues in G_i/o_-coupled 5-HT receptors, including W^6.48^, F^6.51^, F^6.52^, and Y^7.43^. This hydrophobic environment is crucial for ligand-induced receptor activation. Inspection of the binding poses of ligands in G_i/o_-coupled 5-HT receptors reveals that 5-HT, 5-CT, and BRL54443 only occupy the OBP. In contrast, Donitriptan and Lasmiditan, two anti-migraine drugs selectively targeting 5-HT_1B/1D_ and 5-HT_1F_, respectively, are relatively bulky and occupy both OBP and EBP of specific receptors (Supplementary Fig. S4a). These structural observations are consistent with the contention that OBP is critical to the binding potency of ligands, whereas the EBP plays a predominant role in determining ligand selectivity^2^. Together, these findings provide insights into the 5-CT recognition for 5-HT_5A_ and deepen our understanding of ligand selectivity for 5-HT receptors.

### Role of the residue at 6.55 in the determination of 5-CT selectivity for G_i/o_-coupled 5-HT receptors

**Fig. 3:**
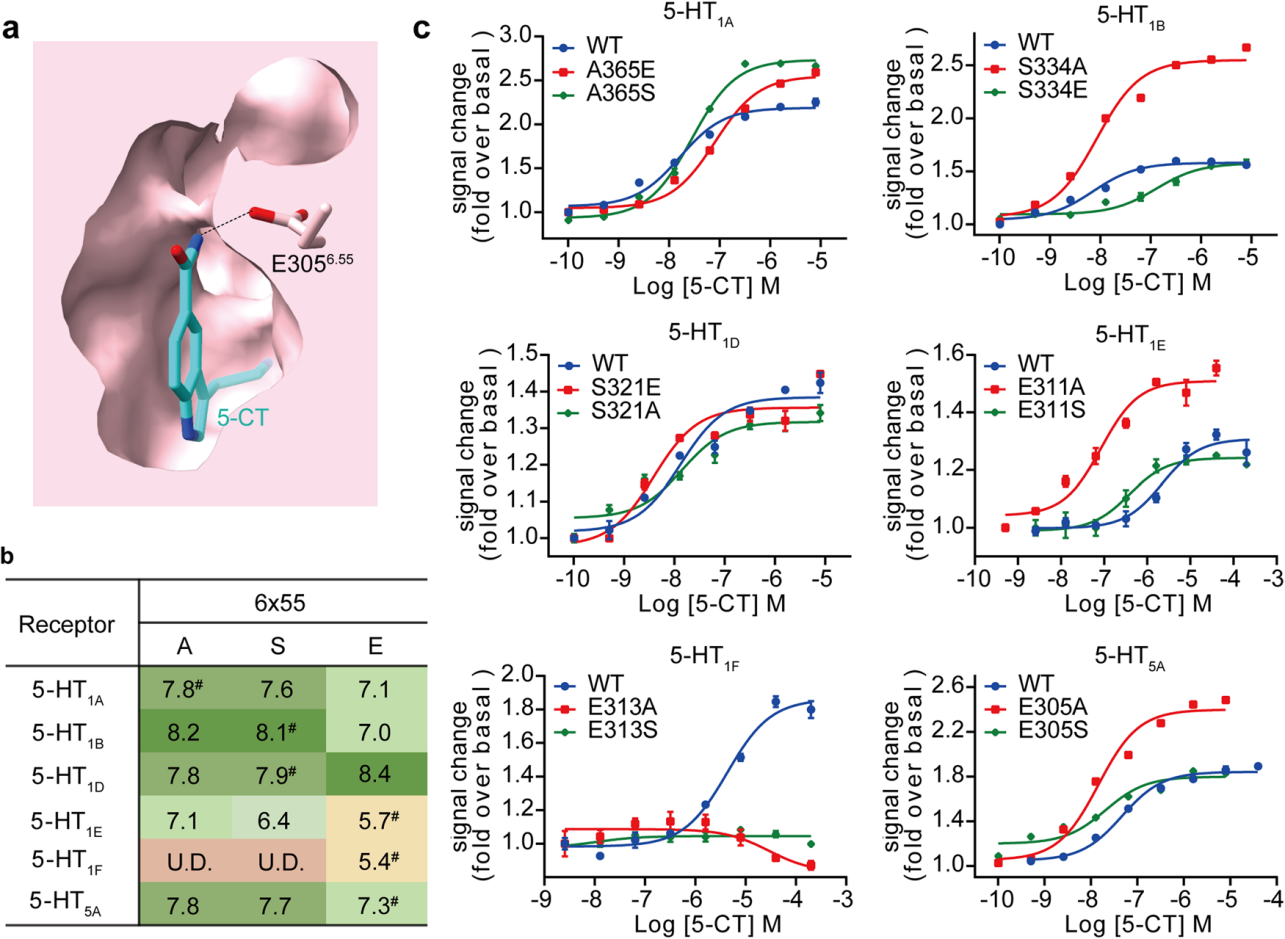
Role of the residue at 6.55 in determining 5-CT selectivity of G_i/o_-coupled 5-HT receptors. **a** Cross-section of the orthosteric binding pocket of 5-HT_5A_. The hydrophobic interaction between 5-CT and E305^6.55^ is indicated as a black dashed line. **b** 5-CT activity (pEC_50_ values) for wild-type (WT) and mutants of G_i/o_-coupled 5-HT receptors. ^#^5-CT activity (pEC_50_ values) for WT receptors. U.D., undetectable. **c** Effects of mutations at 6.55 on 5-CT-induced G_i_ protein recruitment of G_i/o_-coupled receptors. Three independent NanoBiT assays in triplicates were performed. Each data point presents mean ± SEM from a representative experiment.

5-CT shows different selectivity for G_i/o_-coupled 5-HT receptors. It exhibits high affinities for 5-HT_1A_, 5-HT_1B_, and 5-HT_1D_ (*pK*_i_ = 7.9–8.1) and relatively weak affinities for 5-HT_1E_, 5-HT_1F_, and 5-HT_5A_ (*pK*_i_ = 5.4–7.0) (Fig. 3b; Supplementary Table S3). Sequence comparison of residues in the EBP of G_i/o_-coupled 5-HT receptors reveals a low sequence identity at position 6.55 (Supplementary Fig. S4b). The residue at 6.55 is alanine in 5-HT_1A_ and serine in 5-HT_1B_ and 5-HT_1D_. In contrast, the cognate residue in 5-HT_1E_, 5-HT_1F_, and 5-HT_5A_ is glutamic acid. The difference in residue composition raises a hypothesis that the residue at 6.55 is involved in 5-CT selectivity for G_i/o_-coupled 5-HT receptors.

To prove this hypothesis, we introduced swap mutations to residues at 6.55 across G_i/o_-coupled 5-HT receptors. Our G_i_ protein recruitment data support two-facet roles of the residue at 6.55 in 5-CT-induced receptor activation. One is the steric hindrance arising from the side chain of the residue at position 6.55 (Fig. 3c). For 5-HT_1A_, A365^6.55^E and A365^6.55^S mutations, which increase the size of the side chain, reduced 5-CT-induced receptor activation relative to the WT receptor. S334^6.55^E mutation of 5-HT_1B_ decreased 5-CT activity, while substituting the serine with a smaller side chain residue alanine dramatically increased 5-CT potency. Similarly, mutating glutamic acid of 5-HT_1E_ and 5-HT_5A_ to alanine or serine, two residues with a relatively small side chain, notably promoted receptor activation. These findings corroborate the idea that the bulkier side chain of glutamic acid relative to alanine and serine may prevent the binding of 5-CT and receptor activation through steric hindrance. On the other hand, 5-CT may form hydrogen bonds with the glutamic acid at 6.55 in G_i/o_-coupled 5-HT receptors, which may dominate the ligand–receptor interaction over the hindrance effects of the side chain (Fig. 3c). This point is supported by functional analysis of 5-HT_1D_ and 5-HT_1F_. S321^6.55^E mutation of the 5-HT_1D_ enhanced 5-CT activity, despite the increased side-chain size. Consistently, E313^6.55^S and E313^6.55^A mutations in 5-HT_1F_ almost abolished 5-CT activity. Thus, residues at position 6.55 modulate the activity of G_i/o_-coupled 5-HT receptors through two aspects of roles: the steric hindrance effects for 5-HT_1A_, 5-HT_1B_, 5-HT_1E_, and 5-HT_5A_ and the hydrogen bond-forming capacity as an acceptor for 5-HT_1D_ and 5-HT_1F_. Together, our findings provide further evidence for the previous speculation that the residue at 6.55 is responsible for the ligand-recognition specificity of 5-HT receptors and offer a new opportunity for the design of drugs selectively targeting 5-HT receptors^11^.

### General features of the activation and G protein coupling of G_i/o_-coupled 5-HT receptors

**Fig. 4:**
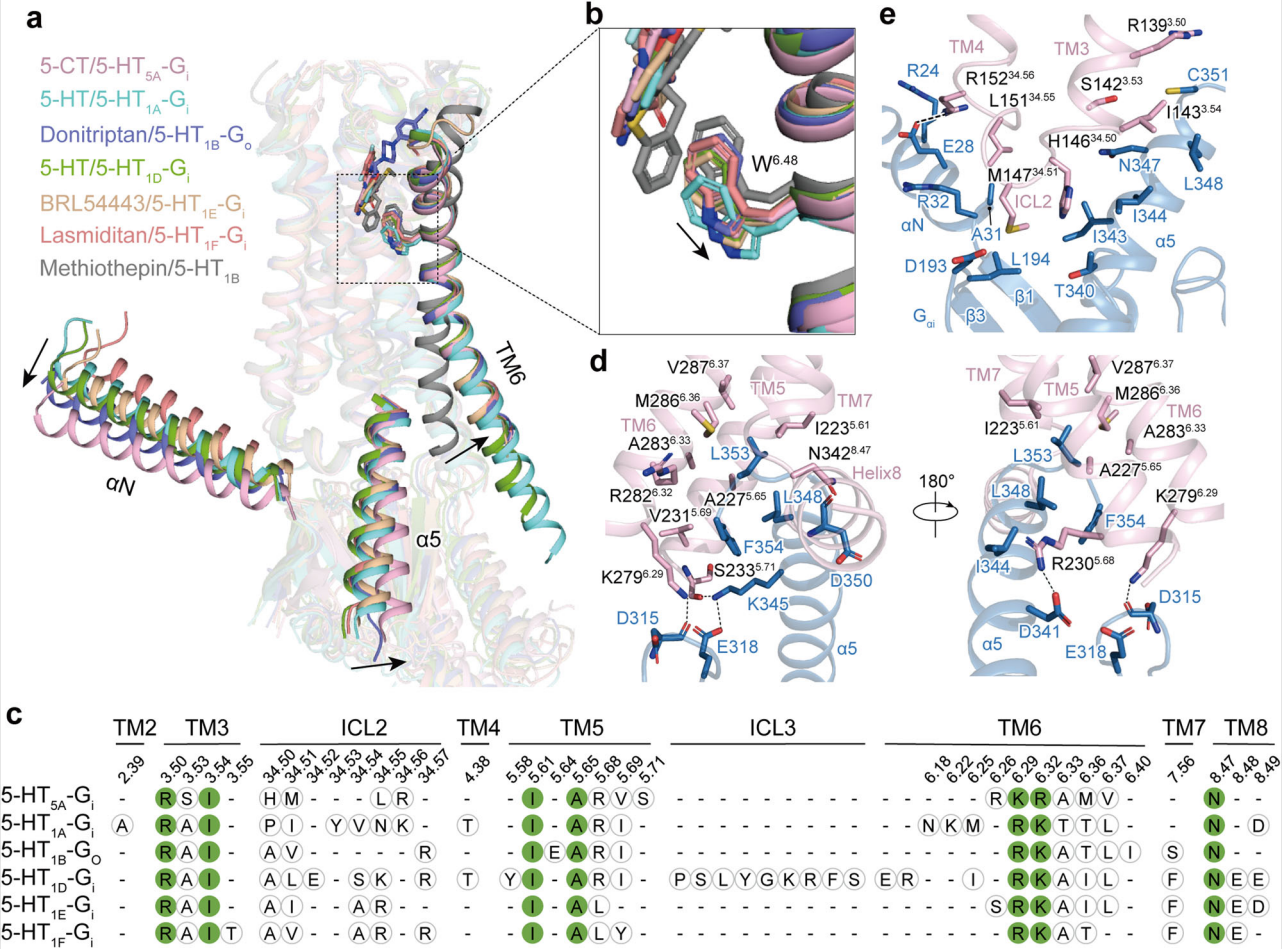
Structural features of the activation and G_i/o_ coupling of 5-HT_5A_ and other G_i/o_-coupled 5-HT receptors. **a** The activation of 5-HT_5A_ and other G_i/o_-coupled 5-HT receptors. The outward movement of TM6 of agonist-bound G_i/o_-coupled 5-HT receptors compared with methiothepin-bound 5-HT_1B_ in the inactive state is shown as a black arrow. The movements of the αN and α5 helix of Gα_i_ protein in the 5-HT_5A_–G_i_ complex relative to that in other G_i/o_-coupled 5-HT receptors are indicated as black arrows. The structures of the active 5-HT_5A_ (light pink), 5-HT_1A_ (cyan, PDB: 7E2Y), 5-HT_1B_ (purple, PDB: 6G79), 5-HT_1D_ (green, PDB: 7E32), 5-HT_1E_ (wheat, PDB: 7E33), 5-HT_1F_ (coral, PBD: 7EXD) and inactive-state 5-HT_1B_ (gray, PDB: 4IAQ) complexes were superimposed based on TM2, TM3 and TM4. **b** Structural comparison of ligands and the W^6.48^ residues of active 5-HT receptors with that of inactive 5-HT_1B_. Ligands directly contact W^6.48^. Compared with methiothepin, the inverse agonist of 5-HT_1B_, agonists trigger the rotameric switch of W^6.48^, which leads to the outward movement of TM6. **c** The sequence alignment of receptor residues at receptor–G_i/o_ protein interfaces. The conserved residues are highlighted in solid green circles. **d**, **e** Detailed interactions between 5-HT_5A_ and the Gα_i_ subunit. Detailed Interactions between TM5, TM6, and the TM7–helix 8 junction of 5-HT_5A_ and the α5 helix of the Gα_i_ subunit (**d**). Detailed interactions between ICL2 of 5-HT_5A_ and the α5 and αN helices, β1 and β3 strands of the Gα_i_ subunit (**e**).

Similar to agonists bound to other G_i/o_-coupled 5-HT receptors, 5-CT directly contacts the toggle switch residue W^6.48^ of 5-HT_5A_ and triggers its rotameric switch. The change of W^6.48^ initiates the rotation and outward movement of the TM6 cytoplasmic end of 5-HT_5A_ relative to inverse agonist-bound 5-HT_1B_ (PDB: 5V54), the hallmark of class A GPCR activation (Fig. 4a, b).

Structural comparison of the G_i_-coupled 5-HT_5A_ with other G_i/o_-coupled 5-HT receptors whose structures had been solved revealed an almost overlapped receptor activation conformation. However, the α5 helices of Gα_i/o_ subunits in these 5-HT receptor complexes showed slight tilts to different extents, which cause rotation of the entire G_i/o_ proteins, leading to the most noticeable translational movement of the αN helices (Fig. 4a). The global coupling interface profile analysis showed that the majority of the Gα_i_ subunit-interacting residues in TM3, ICL2, TM5, TM6, TM7, and helix 8 are conserved across G_i/o_-coupled 5-HT receptors, including R^3.50^, I^3.54^, I^5.61^, A^5.65^, R/K^6.29^, R/K^6.32^, and N^8.47^. Differently, no substantial interactions were seen between ICL3 of G_i/o_-coupled 5-HT receptors and G_i_ protein, with an exception of 5-HT_1D_, which shows an additional EM density of ICL3 and a more extensive ICL3–G_i_ interaction^11^ (Fig. 4c).

Two major interfaces exist between 5-HT_5A_ and G_i_ protein. The cytoplasmic receptor cavity constituted by TM3, TM5, TM6, and the TM7–helix 8 junction accommodates the distal C-terminal end of the α5 helix of the Gα_i_ subunit, forming a primary interface (Fig. 4d). The residues of α5 helix hydrophobically contact the receptor cavity. L348, C351, L353, and F354 in the α5 helix of the Gα_i_ subunit contact a hydrophobic patch comprised of residues in TM3 (I143^3.54^), TM5 (I223^5.61^, A227^5.65^, and V231^5.69^), and TM6 (A283^6.33^ and V287^6.37^). In addition, residues R230^5.68^ and S233^5.71^ of TM5 form well-defined hydrogen bonds with D341 and K345 from the α5 helix, respectively (Fig. 4d). The ICL2 also interacts with Gα_i_, constituting the other major interface. M147^34.51^ of ICL2 inserts into the groove constituted by hydrophobic residues in the α5 helix, the β1 and β3 strands, and αN of the Gα_i_ subunit. A hydrogen bond present between R152^34.56^ and E28 may further stabilize the ICL2–Gα_i_ interface (Fig. 4e). Together, these findings clarify the activation and G_i_ coupling features of 5-HT_5A_ and provide a comprehensive understanding of the G protein coupling mechanism of G_i/o_-coupled 5-HT receptors.

## Discussion

5-HT_5A_ is a G_i/o_-coupled 5-HT receptor subtype and is involved in nervous system disorders, thus serving as an important drug target. It is the only G_i/o_-coupled 5-HT receptor subtype lacking a high-resolution structure to date. In this paper, we report a 3.1 Å-resolution cryo-EM structure of the 5-HT_5A_–G_i_ complex bound to a synthetic agonist, 5-CT, which is also the first 5-CT-bound 5-HT receptor structure. Our structure reveals the recognition mechanism of 5-HT_5A_ by 5-CT and adds to the pool of the structures for deepening our understanding of the ligand-binding mode of 5-HT receptors. Furthermore, structural comparison and functional analysis of the ligand-binding pockets reveal that the residue at 6.55 serves as a determinant for the 5-CT specificity for G_i/o_-coupled 5-HT receptors. This ligand specificity is partly attributed to the steric hindrance arising from the side chain of the residue at 6.55 or its potential polar interaction with ligands. In addition, our structure reveals a similar activation mechanism and an overall conserved G_i_ protein coupling mode for 5-HT_5A_ compared with other G_i/o_-coupled 5-HT receptors. These findings broaden our understanding of ligand recognition in the 5-HT system.

Although 5-HT_5A_ has been cloned for ~3 years, it is still one of the less well-characterized receptors in the 5-HT receptor family. The lack of selective ligands has delayed the functional studies on 5-HT_5A_ until the discovery of the selective antagonists SB699551 and ASP5736, which have improved our understanding of the localization of 5-HT in the brain and its function. However, it should be noted that we are still far from fully understanding the pharmacological characteristics of 5-HT_5A_. Meanwhile, no drugs targeting 5-HT_5A_ have been registered for clinical trials or approved. Recently, virtual screening based on a homology model identified UCSF678, a 42 nM new chemical probe with partial agonism activity for 5-HT_5A_. USCF678 exhibits enhanced selectivity for 5-HT_5A_ and a more restricted off-target profile than the existing 5-HT_5A_ antagonist SB699551. Unlike the promiscuous ligand 5-HT, molecular docking reveals that USCF678 extends into the upper region of the binding pocket, known as EBP. W117^3.28^ in EBP of 5-HT_5A_ is further proved to be responsible for the high-affinity binding of UCSF638, and the tryptophan at position 3.28 may contribute to the off-target binding of UCSF638 analogs^33^. These findings are consistent with the two pockets (OBP and EBP) binding model^2,8^ and highlight the importance of EBP to the discovery of selective ligands. Consequently, the 5-HT_5A_ structure provides an accurate template for the rational design of drugs targeting 5-HT_5A_ and may offer a new opportunity for the treatment of nervous system diseases, including psychosis, depression, schizophrenia, and neuropathic pain.

## Materials and methods

### Constructs

The human full-length 5-HT_5A_ was cloned into the pFastBac with an N-terminal haemagglutinin (HA) sequence followed by a Flag-tag, 15× His-tag, BRIL-tag, and a LgBiT sequence at the C-terminus to facilitate the protein expression and purification. The human Gα_i_ with four dominant-negative mutations, S47N, G203A, E245A, and A326S was applied. Human Gβ_1_, human Gγ_2_, and scFv16 were cloned into pFastBac vector using homologous recombination (ClonExpress One Step Cloning Kit, Vazyme).

### Expression and complex purification

5-HT_5A_-LgBiT, DN_Gα_i_, Gβ_1_-SmBiT, Gγ_2_, and scFv16 were co-expressed in Hi5 insect cells (Invitrogen) using the Bac-to-Bac baculovirus expression system (ThermoFisher). Cell cultures were grown in ESF 921 medium (Expression Systems) to a density of 3 × 10^6^ cells/mL, and then infected with five separate baculoviruses, respectively, at the ratio of 1:1:1:1:1.5. The culture was harvested by centrifugation 48 h post-infection and stored at −80 °C for further usage.

Cell pellets were lysed in 20 mM HEPES, pH 7.4, 20 mM KCl, 10 mM MgCl_2_, 5 mM CaCl_2_, and 10% glycerol supplemented with Protease Inhibitor Cocktail (TargetMol). The 5-HT_5A_–G_i_ complex was formed on the membrane for 1.5 h at room temperature by addition of 10 µM 5-CT and 25 mU/mL apyrase (Sigma), and then solubilized from the membrane by using 0.5% (w/v) *n*-dodecyl-β-d-maltoside (DDM) (Anatrace) and 0.1% (w/v) cholesteryl hemisuccinate (CHS) (Anatrace) for 2 h at 4 °C, followed by centrifugation at 85,000 × *g* for 30 min to extract the solubilized complex. The supernatant was subsequently incubated by nickel affinity chromatography (Ni Smart Beads 6FF, SMART Lifesciences) at 4 °C for 3 h. The resin was washed with 20 column volumes of the buffer containing 20 mM HEPES, pH 7.4, 100 mM NaCl, 25 mM imidazole, 0.01% (w/v) LMNG (Anatrace), 0.005% GDN (Anatrace), 0.004% (w/v) CHS (Anatrace), and 5 µM 5-CT. The complex was then eluted with six column volumes of the same buffer containing 300 mM imidazole. The protein was concentrated and subjected onto a Superdex 200 Increase 10/300 column (GE Healthcare) in the buffer containing 20 mM HEPES, pH 7.4, 100 mM NaCl, 0.00075% (w/v) LMNG (Anatrace), 0.00025% (w/v) GDN (Anatrace), 0.0002% (w/v) CHS (Anatrace) and 10 mM 5-CT. The purified complex fractions were collected and concentrated for cryo-EM experiments.

### Cryo-EM grid preparation and data collection

For the cryo-EM grid preparation, 3 μL of the purified 5-CT–5-HT_5A_–G_i_ complex at a final concentration of 25 mg/mL was applied to glow-discharged holey carbon grids (Quantifoil R1.2/1.3, 300 mesh), and vitrified using a Vitrobot Mark IV (ThermoFisher Scientific) subsequently. Grids were plunge-frozen in liquid ethane using Vitrobot Mark IV (Thermo Fischer Scientific). Frozen grids were transferred to liquid nitrogen and stored for data acquisition. Cryo-EM images were collected by an FEI Titan Krios at 300 kV accelerating voltage equipped with a Gatan K3 Summit direct electron detector at the Center of Cryo-Electron Microscopy Research Center, Shanghai Institute of Materia Medica, Chinese Academy of Sciences (Shanghai, China). A total of 5303 movies were automatically acquired using SerialEM10 in super-resolution counting mode at a pixel size of 1.071 Å. The images were recorded at a dose rate of about 26.7 e/Å^2^/s with a defocus ranging from –1.2 to –2.2 μm. The total exposure time was 3 s, and intermediate frames were recorded in 0.083-s intervals, resulting in a total of 36 frames per micrograph.

### Image processing and 3D reconstruction

Image stacks were subjected to beam-induced motion correction using MotionCor2.1^34^, while contrast transfer function (CTF) parameters were determined by Gctf^35^. Automated particle selection and data processing were performed using Relion 3.0^36^. Automated particle selection yielded 3,767,450 particles. The particles were subjected to reference-free 2D classification, producing 1,327,660 particles with well-defined averages. The map of 5-HT_1E_–G_i_–scFv16 complex (EMDB-30975)^11^ low-pass filtered to 40 Å was used as an initial reference model for 3D classification, which produced two good subsets showing clear structural features accounting for 754,854 particles. These particles were subsequently subjected to Bayesian polishing, CTF refinement, and 3D refinement, which generated a map with an indicated global resolution of 3.1 Å at a Fourier shell correlation of 0.143. Local resolution was determined using the Resmap^37^ with half maps as input maps.

### Model building and refinement

The cryo-EM structure of the 5-CT–5-HT_5A_–G_i_ complex (PDB: 7E2Y) and the G_i_ protein model (PDB: 6DDE) were used to generate the initial model and refinement against the electron microscopy map. The model was docked into the EM density map using UCSF Chimera^38^, followed by iterative manual adjustment and rebuilding in COOT^39^ and ISOLDE^40^ according to side-chain densities. Real-space refinement was performed using Phenix programs^41^. The model statistics were validated using MolProbity^42^. Structural figures were prepared in Chimera, ChimeraX^43^, and PyMOL (https://pymol.org/2/). The final refinement statistics are provided in Supplementary Table S1.

### NanoBiT G protein recruitment assay

NanoBiT, a NanoLuc luciferase-based method, is used to detect the interaction between receptor and G protein in living cells^44^. The full-length 5-HT_5A_ was fused with a LgBiT fragment (17.6 kDa) at its C-terminus via a 15-amino acid flexible linker. SmBiT, a 13-amino acid peptide, was C-terminally fused to the Gβ subunit using the same linker. The cDNAs of 5-HT_5A_-LgBiT, Gα_i1_, Gβ_1_-SmBiT, and Gγ_2_ were cloned into pFastBac vector (Invitrogen). The baculoviruses were prepared using the bac-to-bac system (Invitrogen). Hi5 cells were cultured in ESF 921 medium (Expression Systems) to a density of 2.5–3 million cells per mL and then infected with four separate baculoviruses at the ratio of 1:1:1:1. After 48 h infection, the culture was collected by centrifugation, and the cell pellet was resuspended with PBS. The cell suspension was seeded onto 384-well microtiter plates (40 μL per well) and loaded with 5 μL of 50 μM coelenterazine (Yeasen) diluted in the assay buffer. 5 μL of ligands were added and incubated for 3–5 min at room temperature before measurement. Luminescence counts were normalized to the initial count to show the G protein binding response.

### Surface expression analysis

Cell surface expression for each mutant was monitored using flow cytometry. The expressed Hi5 cells (10 μL) were incubated with 10 μL anti-FLAG-FITC antibody (Sigma), which is diluted with PBS containing 4% BSA at a final ratio of 1:1000, at 4 °C for 15 min, and 180 μL 1× PBS was then added to the cells. The surface expression of each mutant was monitored by detecting the fluorescent intensity of FITC using a BD ACCURI C6.

## Supplementary information

Supplementary Information

## Data Availability

The atomic coordinate and the electron microscopy map for the 5-CT–5-HT_5A_–G_i_–scFv16 complex have been deposited in the Protein Data Bank (PDB) under accession number 7X5H and Electron Microscopy Data Bank (EMDB) under accession number EMD-33014, respectively.
