# Supplementary material for: Structural insights into the ligand binding and Gi coupling of serotonin receptor 5-HT5A
Source: Cell Discov. 2022 May 24;8:50. doi: 10.1038/s41421-022-00412-3 (PMC9130316; doi:10.1038/s41421-022-00412-3)
Supplement: Supplementary file 1 — Supplementary Information [file 41421_2022_412_MOESM1_ESM.pdf]

Supplementary Information

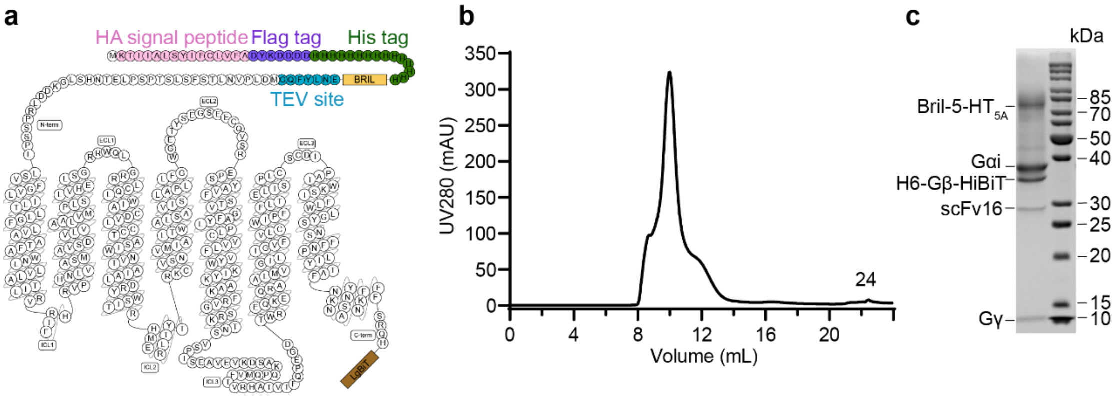

Fig. S1 Characterization and purification of the 5-CT–5-HT$_{5A}$–G$_{i}$ complex. a Schematic illustration of the HA–Flag–His15–BRIL–5-HT$_{5A}$–TEV site–LgBiT construct used in the study. The HA signal peptide (pink), Flag (purple), His15 (green), BRIL (yellow), TEV (blue) and LgBiT (brown) are indicated. **b** Analytical size-exclusion chromatography of the purified complex on Superdex 200 Increase 10/300 column. **c** SDS-PAGE analysis of the purified complex with Coomassie blue staining.

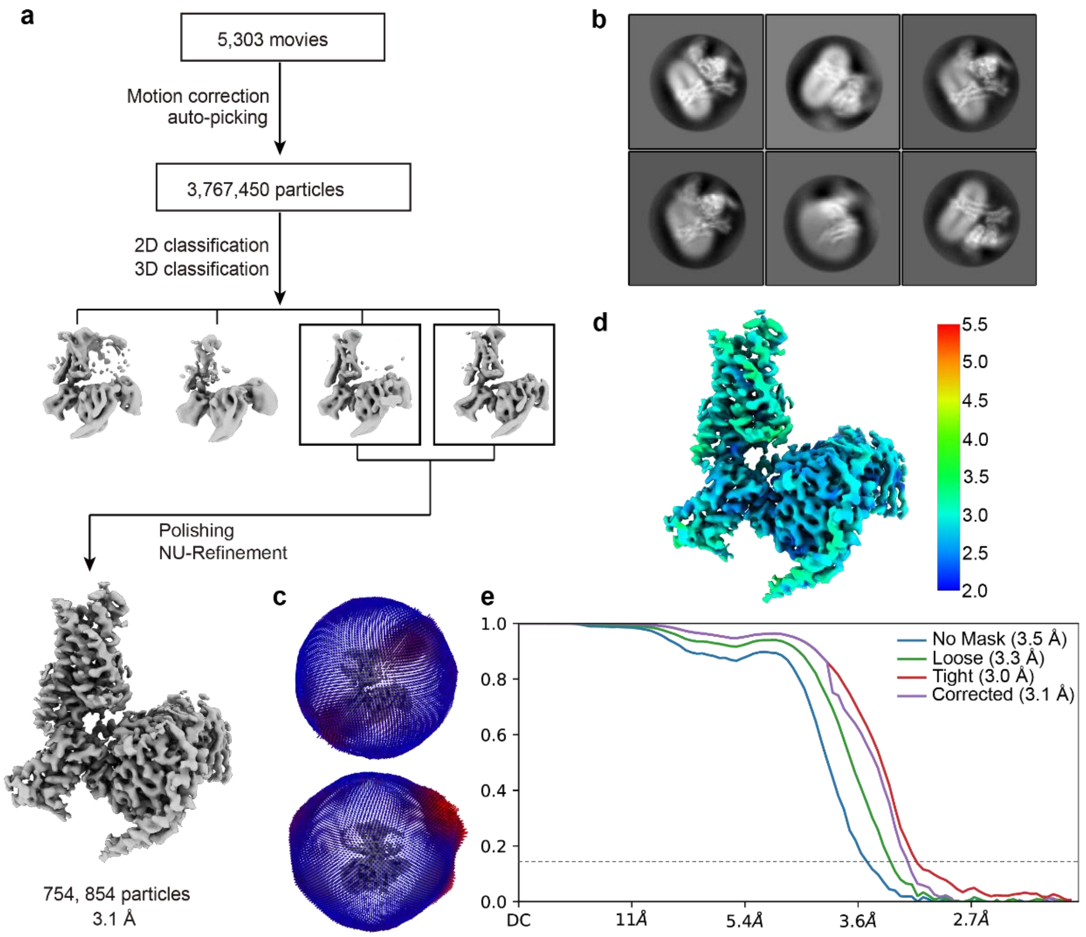

Fig. S2 The cryo-EM data processing and density map of the 5-CT–5-HT$_{5A}$–G$_i$ complex. **a** Flow chart of cryo-EM data processing. **b** Representative cryo-EM micrograph from 5,303 movies and representative two-dimensional class averages determined using approximately 3.8 million particles of the 5-CT–5-HT$_{5A}$–G$_i$ complex were shown. **c** Angle distribution maps. **d** Cryo-EM map of the 5-CT–5-HT$_{5A}$–G$_i$ complex, colored by local resolution (Å) calculated using Resmap package. **e** 'Gold-standard' Fourier shell correlation curves of the 5-CT–5-HT$_{5A}$–G$_i$ complex.

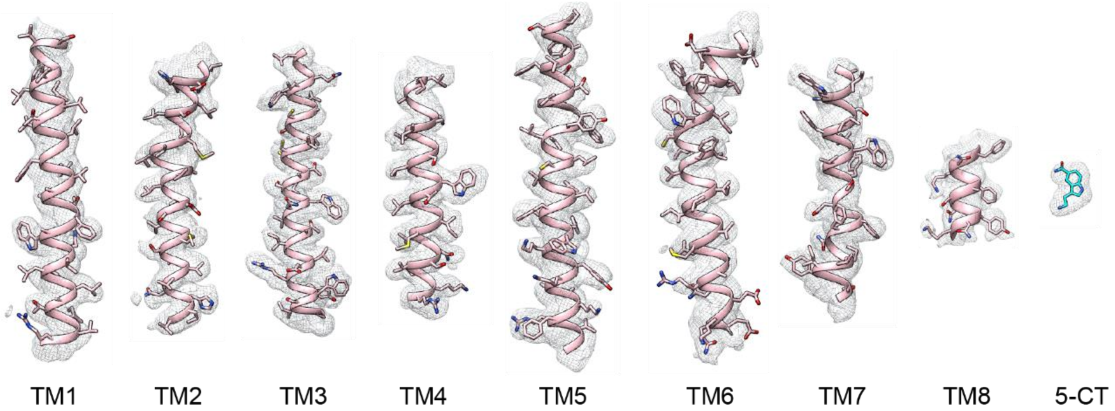

Fig. S3 Representative cryo-EM density map of the 5-CT–5-HT$_{5A}$–G$_i$ complex. Cryo-EM density of transmembrane helices, helix 8 and 5-CT.

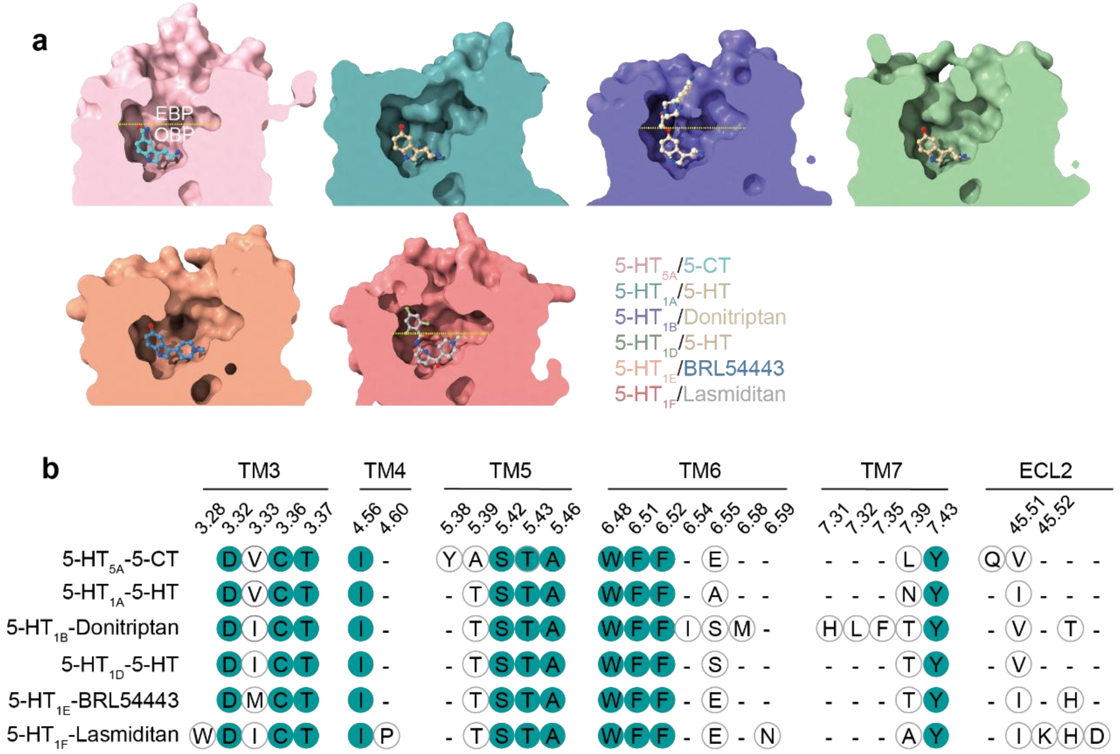

Fig. S4 Comparison of the binding pose of agonists and sequence alignment of residues in the binding pockets of $G_{i/o}$-coupled 5-HT receptors. a Comparison of the agonist-binding poses in $G_{i/o}$-coupled 5-HT_{5A} (light pink), 5-HT_{1A} (cyan, PDB code: 7E2Y), 5-HT_{1B} (purple, PDB code: 6G79), 5-HT_{1D} (green, PDB code: 7E32), 5-HT_{1E} (wheat, PDB code: 7E33), and 5-HT_{1F} (coral, PBD code: 7EXD). **b** Sequence alignment of residues in the ligand-binding pocket of $G_{i/o}$-coupled 5-HT receptors. The conserved residues are highlighted in solid dark green circles.

Table S1. Cryo-EM data collection, model refinement, and validation statistics

	5-HT-5-HT$_{5A}$–G$_i$ (PDB: 7X5H, EMD-33014)
**Data collection and processing**	
Magnification	46,685
Voltage (kV)	300
Electron exposure (e$^-$/Å^2)	70
Defocus range (μm)	-1.2 ~ -2.2
Pixel size (Å)	1.071
Symmetry imposed	C1
Initial particle projections (no.)	3,767,450
Final particle projections (no.)	754,854
Map resolution (Å)	3.1
FSC threshold	0.143
Map resolution range (Å)	2.5-5.0
**Refinement**	
Initial model used	7E2Y
Model resolution (Å)	3.3
FSC threshold	0.5
Map sharpening *B* factor (Å^2)	159.5
Model composition	
Non-hydrogen atoms	8768
Protein residues	1129
Lipid	0
Water	0
B factors (Å^2)	
Protein	151.3
Ligand	85.6
R.m.s. deviations	
Bond lengths (Å)	0.003
Bond angles (°)	0.882
Validation	
MolProbity score	1.21
Clashscore	4.27
Rotamer outliers (%)	0.00
Ramachandran plot	
Favored (%)	98.38
Allowed (%)	1.62
Disallowed (%)	0

Table S2. Effects of alanine mutations of residues in the 5-HT$_{5A}$ pocket on the 5-CT-induced G$_i$ recruitment. The NanoBiT assay was used to evaluate the G$_i$-coupling activity of wild-type 5-HT$_{5A}$ and its mutants. Data shown are means ± S.E.M. of three independent experiments, conducted in triplicate. All data were analyzed by ordinary one-way ANOVA with Dunnett's test. $^{*}P$<0.05, $^{**}P$<0.01; $^{***}P$<0.001, *vs.* WT receptor. U.D., undetectable.

**Const.**	***pEC$_{50}$*** **± S.E.M.**	**Fold over basal (Mean ± S.E.M.)**	**Cell surface expression(%WT)**
WT	7.331 ± 0.047	2.218 ± 0.003	100
D121A	U.D.	1.111 ± 0.012	208.25
V122A	4.952 ± 0.076***	2.426 ± 0.067	187.97
C125A	5.643 ± 0.048***	2.627 ± 0.026	78.08
V194A	6.524 ± 0.057***	2.098 ± 0.021	68.79
S204A	6.097 ± 0.072*	2.090 ± 0.013	79.05
T205A	5.790 ± 0.128***	2.401 ± 0.044	88.33
W298A	7.013 ± 0.104	1.167 ± 0.010	131.16
F301A	6.210 ± 0.104***	2.066 ± 0.019	145.33
F302A	7.066 ± 0.075	1.728 ± 0.041	139.10
E305A	7.838 ± 0.026**	2.399 ± 0.008	102.65
L324A	7.009 ± 0.124	1.537 ± 0.019	79.39
Y328A	4.614 ± 0.053***	1.070 ± 0.009	110.59

Table S3. Effects of mutation of residue at 6.55 on the 5-CT-induced $G_{i/o}$ recruitment. *pEC_{50}* values were determined using a three-parameter logistic equation. Data shown are means ± S.E.M. of three independent experiments, conducted in triplicate. Cell surface expression values were normalized to the wild-type (WT), which was set to 100%. Ordinary one-way ANOVA with Dunnett's test was used to determine the *P* values. $^{*}P<0.05$; $^{**}P<0.01$; $^{***}P<0.001$ *vs.* WT receptor. U.D., undetectable.

Receptor	Mutants	*pEC_{50}* ± S.E.M.	Fold over basal (Mean ± S.E.M.)	Cell surface expression (%WT)
5-HT_{5A}	WT	7.331 ± 0.063	1.834 ± 0.003	100
	E305A	7.838 ± 0.026**	2.397 ± 0.013	102.65
	E305S	7.686 ± 0.082*	1.812 ± 0.003	114.30
5-HT_{1A}	WT	7.787 ± 0.082	2.190 ± 0.025	100
	A365E	7.093 ± 0.032***	2.551 ± 0.024	148.36
	A365S	7.553 ± 0.033*	2.734 ± 0.003	107.20
5-HT_{1B}	WT	8.160 ± 0.091	1.580 ± 0.006	100
	S334E	6.951 ± 0.177***	1.574 ± 0.036	75.20
	S334A	8.054 ± 0.014	2.550 ± 0.008	86.33
5-HT_{1D}	WT	7.859 ± 0.093	1.386± 0.012	100
	S321E	8.408 ± 0.032*	1.356 ± 0.005	83.39
	S321A	7.823 ± 0.131	1.319± 0.004	114.37
5-HT_{1E}	WT	5.660 ± 0.034	1.308 ± 0.031	100
	E311A	7.105 ± 0.180**	1.510 ± 0.026	136.07
	E311S	6.400 ± 0.086**	1.243± 0.003	102.91
5-HT_{1F}	WT	5.365 ± 0.056	1.863 ± 0.032	100
	E313A	U.D.	0.823 ± 0.024	94.03
	E313S	U.D.	1.031 ± 0.023	104.70
